# Problematic social media use is associated with believing in and engaging with fake news

**DOI:** 10.1371/journal.pone.0321361

**Published:** 2025-05-07

**Authors:** Dar Meshi, Maria D. Molina

**Affiliations:** Department of Advertising and Public Relations, Michigan State University, East Lansing, Michigan, United States of America; National Research Council (CNR), ITALY

## Abstract

Social media use is ubiquitous in our modern society, and some individuals display excessive, maladaptive use of these online platforms. This problematic social media use (PSMU) has been associated with greater impulsivity and risk-taking. Importantly, studies in healthy individuals have demonstrated that greater cognitive impulsivity is associated with a greater susceptibility to online “fake news.” Therefore, we hypothesized that PSMU would be associated with believing in and engaging with fake news. To address this, we conducted an online, within-subject experiment in which participants (N=189; female=102, male=86, prefer not to disclose=1; mean age=19.8 years) completed a fake news task. This task presented participants with 20 news stories (10 real and 10 false, in random order) formatted as social media posts. We assessed participants’ credibility judgments of these news posts, as well as participants’ intentions to click, like, comment, and share these posts. We also assessed participants’ degree of PSMU and then related this measure to their performance in our task. We conducted a repeated measures analysis of variance (ANOVA) with a mixed model approach, and it revealed that the greater one’s PSMU, the more one finds specifically false news credible. We also found that the greater one’s PSMU, the greater one’s engagement with news posts, agnostic to the type of content (real or false). Finally, we found that the greater one’s PSMU, the greater one’s intent to click on specifically false news. Our research demonstrates that individuals who experience the most distress and impairment in daily functioning from social media use are also the most susceptible to false information posted on social media. We discuss the clinical implications of our findings.

## Introduction

Almost five billion people around the world, including 82% of Americans, currently use social media [1,2]. These online platforms, like Instagram, X (formerly Twitter), and Facebook, allow individuals to create profiles and post content (e.g., text, images, videos), as well as observe and interact with others. For example, social media users can “like” and comment on each other’s posts. As a result, individuals who use social media receive myriad social rewards [3]. These social rewards act as reinforcers, bringing people back to these sites repeatedly and for substantial durations of time [4] – users worldwide report spending an average of over two hours a day on social media [5]. Importantly, some individuals use social media so much that it becomes maladaptive and these people experience distress and/or impairment in daily functioning [6]. This excessive and maladaptive social media use is termed problematic social media use (PSMU).

PSMU can mirror substance use and other behavioral addictive disorders in some ways [6]. For example, individuals displaying PSMU experience similar symptoms – they may experience withdrawal if they don’t have access to social media, and if they attempt to quit, they may relapse to use social media again. PSMU has also been linked to job loss, poor academic performance, and poor mental health [7–9]. To note, however, the American Psychiatric Association’s Diagnostic and Statistical Manual of Mental Disorders, Fifth Edition, Text Revision [10] does not currently recognize PSMU use as a clinical disorder, and the inclusion of an official diagnosis in the future has been debated in the literature [11,12]. Nevertheless, the U.S. Surgeon General recently released a report highlighting youth’s potential risk of harm from excessive and problematic use of social media [13]. Regarding prevalence, a recent study examined PSMU in 222,532 adolescents across 44 countries revealing a rate of around 7% [14], while a previous meta-analysis of 63 studies across 32 countries revealed a rate of around 5% in young adults [15]. Similar to substance use and other behavioral addictive disorders, research on PSMU has also revealed aberrations in decision making. For example, multiple studies have demonstrated that PSMU is associated with greater general impulsivity, attentional impulsivity, and impulsive choice (for review see [16]). In particular, several studies have demonstrated that PSMU is associated with greater impulsivity using a measure of delay discounting [17] and greater risk-taking with other related decision-making tasks [18,19] (although see [20]). With these increased aspects of impulsivity and risk-taking in mind, we considered another important societal issue concerning social media use, the consumption and spread of “fake news” online.

Fake news – also termed false news – can be defined as “fabricated information that mimics news media content in form but not in organizational process or intent” [21]. False news is termed “misinformation” if the false information is unintentionally distributed, and “disinformation” if the false information is intentionally distributed to deceive people [21]. The distribution of false news has played an important role in society throughout history. For example, the British used radio broadcasts to spread disinformation in Germany and undermine the Nazi party during World War II [22]. More recently, false news has become a hotly debated and researched topic because of its ability to be shared and spread over social media. For example, over 60% of people in the US read news content on social media [23], and research has demonstrated that false news disseminates over social media at a greater rate than real news [24]. Importantly, false news spread over social media can have major effects, such as influencing the US presidential elections in 2016 [25]. Researchers have tried to better understand the cognitive processes involved when individuals find false news credible and engage with it by sharing it on social media (for review see [26]). Several studies used a cognitive reflection task (CRT) to demonstrate that both susceptibility to false news and sharing of false news on social media are associated with an impulsive lack of careful reasoning [27–29]. Individuals who score low on the CRT are thought to be “cognitively impulsive” – for example, these individuals demonstrate greater impulsivity in a measure of delay discounting [30]. In the false news context, the CRT is thought to measure one’s reflexive impulsivity, defined as one’s “tendency to unthinkingly accept incoming information as being valid and true” [28].

Taken together, the above-described research indicates that PSMU is associated with several aspects of impulsivity, including impulsivity as measured by delay discounting, and low scores on the CRT have been associated with both impulsivity as measured by delay discounting and believing in and spreading false news. Therefore, we hypothesized that greater PSMU is associated with poorer credibility judgments of false news, as well as greater engagement with false news. No study had previously addressed this gap in the literature, so we conducted an online experiment in which participants completed a fake news task, modeled after previous studies [27,28,31]. This task assessed participants’ credibility judgments of news articles presented as social media posts, as well as participants’ intentions to click, like, comment, and share these posts. Of note, the above-mentioned research that examined the relationship between the CRT and fake news typically assessed only news story credibility and intent to share these stories on social media [27–29], however, other studies have examined interactions with fake news with respect to clicks, likes, and comments, as well as shares [32–34]. Furthermore, understanding whether PSMU is associated with greater engagement with false news over multiple measures (clicks, likes, comments, shares) is also important from a virality point of view. That is, the more users engage with such content, the more viral it becomes, increasing the effects of false information through these online platforms. Therefore, we collected all these measures of engagement (clicks, likes, comments, shares) in our task. Finally, we also assessed participants’ degree of PSMU and then related this measure to their performance in the fake news task.

## Methods

### Participants

Undergraduate students at a large U.S. university were recruited through an online student pool and participated for course credit. To take part in our study, individuals were required to be at least 18 years of age and self-report that they used at least one social media platform. Our sample size consisted of 189 participants (female=102, male=86, prefer not to disclose=1) after excluding: 17 participants who indicated that they did not take the survey seriously and/or failed our attention check (see below), and one participant whose responses on two dependent variables were more than three standard deviations from the sample mean. The average age of our final sample was 19.8 years (*SD*=1.49, range=18–26), and the majority of our sample, 68.8% (n=130), was white, while 31.2% (n=59) was non-white (American Indian, Asian, black, Native Hawaiian, multi-race).

### Procedure

We conducted a fully online, within-subject experiment. Participants first provided informed, electronic consent and then answered demographic questions. Next, they completed our fake news task and then answered questions about their problematic social media use. All procedures were approved by the university’s Institutional Review Board (STUDY00005421), and all data were collected in December 2020 (data are available at the Open Science Framework repository: https://osf.io/7fngw).

### Fake news task

All participants performed 20 trials of a fake news task, modeled after previous work by Pennycook and colleagues [27,28,31]. Participants were randomly presented with 10 news stories deemed false by an independent fact‐checker (snopes.com), and 10 news stories published on the websites of news organizations (e.g., apnews.com, wsj.com, nytimes.com, reuters.com) deemed to be reliable by an independent media organization [35]. Hence, all participants experienced two conditions (false news and real news). These news stories were presented in the format of a social media post, showing the participant an image, the headline, the beginning of the article, and the web address of the publisher (the full stimulus set is available at the Open Science Framework repository: https://osf.io/7fngw). All news stories were non-political and varied in the topics they covered. Of note, we did not balance topics between conditions due to concern that study participants, upon seeing two stories on the same topic, would be able to decipher the study manipulation (real vs. fake news). In addition, we did not balance sources (web addresses) between conditions, as we wanted participants to be able to use the source of the news story in their credibility judgment, as this maintains the face validity of our manipulation. While viewing the post, participants answered seven questions about the news article.

The first three questions concerned the post’s credibility, modeled after previous research obtaining message credibility [36]. Participants were asked to “Please rate how well each of the following adjectives describes the above news story,” and were then provided with three adjectives: 1. Accurate, 2. Authentic, and 3. Believable. For each adjective, participants used a 5-point scale to respond (1=“not well”; 5=“very well”). Item ratings from each participant were averaged for the 10 fake news posts (*M*=2.82, *SD*=0.05, Cronbach’s α=0.95) and for the 10 real news posts (*M*=3.48, *SD*=0.05, Cronbach’s α=0.97) to obtain each participant’s mean perception of credibility for both types of posts.

The last four questions concerned participants’ potential engagement with the post. Participants were asked to “Please imagine that you came across the above news story while scrolling through content posted on social media (e.g., on Facebook, Twitter, etc.),” and were then asked to rate the likelihood that they would take each of the following four actions: 1. Click on the post, 2. Like the post, 3. Comment on the post, and 4. Share the post. For each action, participants used a 5-point scale to respond (1=“not at all likely”; 5=“very likely”). For analyses, item ratings from each participant were averaged for both the 10 fake news posts and the 10 real news posts. Please see Table 1 which reports sample means and standard deviations for each engagement question.

**Table 1:** Participant mean performance and comparison across type of content condition in the fake news task.

Variable	Fake News Items	Real News Items	*F*-value
Credibility Rating	2.82 (.05)	3.48 (.05)	199.23^***^
Likelihood to:			
Click	2.74 (.07)	2.41 (.07)	41.21^***^
Like	2.01 (.07)	1.99 (.07)	0.47
Comment	1.71 (.06)	1.66 (.06)	3.55
Share	2.17 (.06)	1.83 (.06)	57.37^***^

Note: Data presented are means with standard error in parentheses; degrees of freedom for each comparison = 187.

* *p* <0.05,

** *p* <0.01,

*** *p* <0.001.

### Problematic social media use

Problematic social media use was measured with the 6-item Bergen Social Media Addiction Scale [37,38]. The reliability and validity of this scale has been established [38], and it is one of the most widely used scales to assess PSMU [39]. Participants were prompted with “Please answer the following questions with regard to your social media use over the past year” and each item assessed a commonly accepted core aspect of addiction: preoccupation, mood modification, tolerance, conflict, withdrawal, and relapse [6]. For example, the item concerning withdrawal asked, “Do you become restless or troubled if you are prohibited from using social media?” Participants responded on a 5-point scale (1=”very rarely”; 5=”very often”), and we summed responses to compute a single score for each participant (*M*=15.10, *SD*=5.49, range=6–29, Cronbach’s α=0.90).

### Attention check and survey seriousness

The survey included an attention check of four questions distributed across the questionnaire. Regarding the structure of these questions, each attention item consisted of a direct question stem with a dichotomous response option. All participants should have known the answers to these questions, which assessed, for example, participants’ knowledge of the city where their university is located. At the end of the survey, we also asked each participant whether they had “taken part in this survey seriously.” They were told that their response to this question would not affect the course credit they would receive for participating. Participants were given two response options: “I have taken part seriously” and “I have just clicked through, please throw my data away”. The 17 participants who failed one of our attention checks by answering incorrectly or indicated that we should not use their data were excluded from analysis.

### Data analysis

To assess if participants’ responses in the fake news task differed as a function of their PSMU and the type of content (false or real news posts), we employed JMP statistical software (SAS Institute, Inc., Cary, NC) and ran repeated measures analyses of variance (ANOVA) using a mixed model approach. A repeated measure analysis was necessary, given that all participants were exposed to both real and false news. Thus, the type of content was a within-subject variable. Furthermore, the benefit of using a repeated measures analysis with a mixed model approach is that, in comparison to other linear models, subject-specific random effects are accounted for and considered residual effects. This means that individual differences and demographic variables are accounted for and do not influence our results. Specifically, our variables were entered in the model accordingly – we entered the type of content (dichotomous variable) and PSMU (continuous variable) as independent variables and participant ID as a random effect. We ran one analysis for each of our five dependent variables of interest (credibility, intention to click, intention to like, intention to comment, and intention to share).

## Results

Please see Table 1 for overall performance in our fake news task with respect to type of content (fake or real). Participants rated real news articles to be more credible than fake news articles (*F*_(1, 187)_=199.23, *p*<0.001). Participants also reported a greater intention to click (*F*_(1, 187)_=41.21, *p*<0.001) and share (*F*_(1, 187)_=57.37, *p*<0.001) fake news posts in comparison to real news posts. Participants reported no difference in intention to like (*F*_(1, 187)_ =0.047, *p*>0.05) or comment (*F*_(1, 187)_=3.55, *p*>0.05) on fake news posts in comparison to real news posts. Please see Table 2 for correlations between all variables of interest.

**Table 2:** Correlation table for all variables of interest.

Variable	1	2	3	4	5	6	7	8	9	10
1. Credibility Fake	1									
2. Credibility Real	0.477^**^	1								
3. Click Fake	0.506^**^	0.283^**^	1							
4. Click Real	0.354^**^	0.332^**^	0.712^**^	1						
5. Like Fake	0.468^**^	0.076	0.647^**^	0.602^**^	1					
6. Like Real	0.348^**^	0.086	0.572^**^	0.729^**^	0.857^**^	1				
7. Comment Fake	0.315^**^	−0.036	0.509^**^	0.535^**^	0.762^**^	0.753^**^	1			
8. Comment Real	0.237^**^	−0.066	0.412^**^	0.558^**^	0.678^**^	0.813^**^	0.883^**^	1		
9. Share Fake	0.443^**^	0.076	0.690^**^	0.511^**^	0.785^**^	0.690^**^	0.724^**^	0.622^**^	1	
10. Share Real	0.307^**^	0.021	0.548^**^	0.674^**^	0.751^**^	0.883^**^	0.814^**^	0.882^**^	0.764^**^	1
11. PSMU	0.192^**^	0.001	0.237^**^	0.047	0.238^**^	0.172^*^	0.251^**^	0.206^**^	0.248^**^	0.172^*^

Note:

* *p* <0.05,

** *p* <0.01.

**Fig 1:**
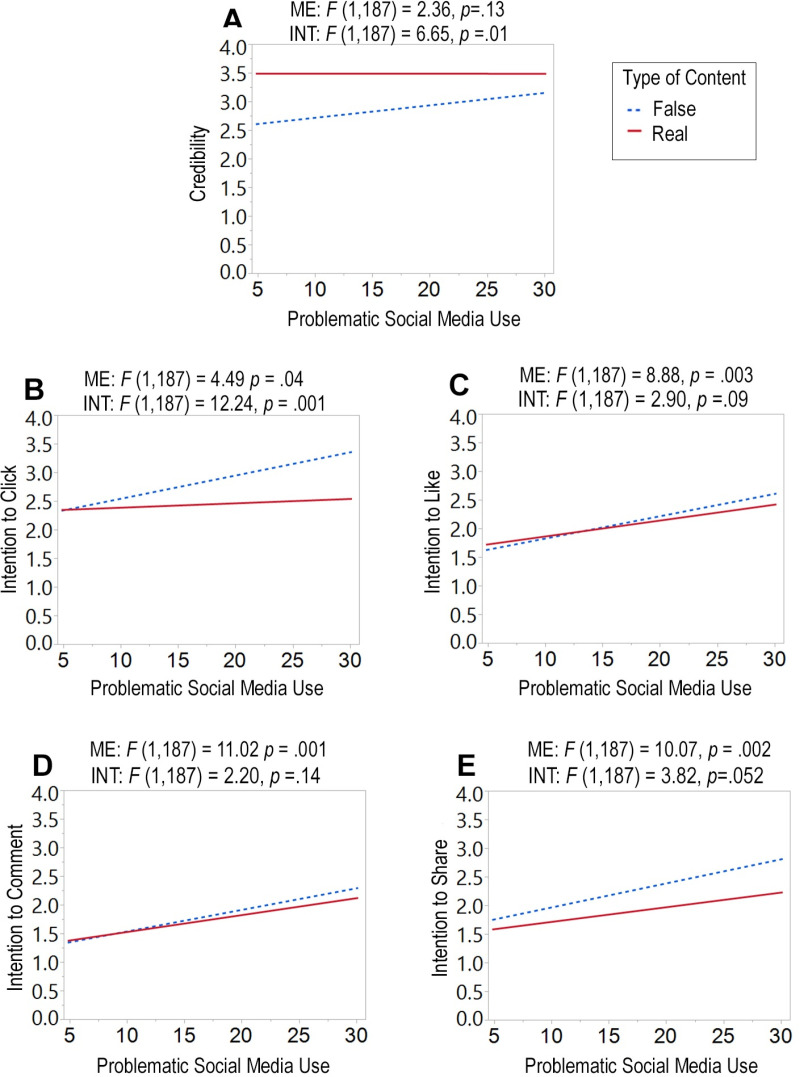
Task performance as a function of problematic social media use. Data displayed with respect to (A) credibility, (B) likelihood to click on a news post, (C) likelihood to like a news post, (D) likelihood to comment on a news post, and (E) likelihood to share a news post. Reported statistics above plots are main effect (ME) of problematic social media use and interactions (INT) between problematic social media use and type of content (fake vs. real) with respect to these five different measures.

We also examined whether PSMU is associated with overall credibility ratings and engagement with social media news posts, agnostic to the type of content (Fig 1). We revealed no main effect of PSMU with credibility ratings (*F*_(1, 187)_=2.36, *p*=0.13). In other words, there was no relationship between PSMU and overall perceptions of credibility of news articles. However, we revealed significant main effects with intention to click (*F*_(1, 187)_=4.49, *p*=0.04), intention to like (*F*_(1, 187)_=8.88, *p*=0.003), intention to comment (*F*_(1, 187)_=11.02, *p*=0.001), and intention to share (*F*_(1, 187)_=10.07, *p*=0.002). This demonstrates that the greater one’s PSMU, the more likely one is to engage with a news article posted on social media, regardless of the type of article (fake or real), across all four of our measures.

In addition, we examined interaction effects to reveal if PSMU is associated with the type of content (fake or real news posts) across our dependent measures. Our analysis revealed a significant interaction between PSMU and type of content with respect to credibility (*F*_(1, 187)_=6.65, *p*=0.01). This effect is driven by fake news content (Fig 1A), as our data demonstrate that the greater one’s PSMU, the greater one believes that fake news is credible. Conversely, there was no observable relationship between PSMU and perceived credibility of real news posts. Our analysis also revealed a significant interaction between PSMU and type of content with respect to intention to click (*F*_(1, 187)_=12.24, *p*=0.001), and a near significant interaction for intention to share (*F*_(1, 187)_=3.82, *p*=0.052). Again, these effects were driven by fake news content (Figs 1B and 1E, respectively), demonstrating that the greater one’s PSMU, the greater one’s intention to click and share fake news content. Conversely, for real news, there were no observable relationships between PSMU and intentions to click and share. Finally, our analysis did not reveal significant interaction effects between PSMU and type of content with respect to intention to like (*F*_(1, 187)_=2.90, *p*=0.09, Fig 1C) and intention to comment (*F*_(1, 187)_=2.20, *p*=0.14, Fig 1D).

## Discussion

We assessed participants’ PSMU and related it to their performance in a fake news task. This task assessed participants’ credibility judgements of, and engagement with, both false and real news articles which appeared to have been posted to social media. Our study yielded several notable findings. To begin with, our analyses revealed that participants found real news articles more credible than false news articles, as expected. We also found that people engage more with false news in regard to clicking and sharing, but not liking and commenting. This somewhat replicates previous research. For example, Edelson and colleagues used data from Facebook to demonstrate that people react (“like”), comment, and share misinformation more than non-misinformation [40], while Vosoughi and colleagues used data from the former Twitter platform to also demonstrate that false news is shared more often than real news [24].

In regard to our primary research question, we examined the relationship between PSMU and performance in our fake news task. Our study revealed no relationship between PSMU and credibility judgments of overall news posts, agnostic to content type, but we did find a significant relationship between PSMU and credibility judgments of specifically false news posts. As hypothesized, the greater one’s PSMU, the more one finds false news credible. We also found relationships between PSMU and engagement with overall news posts. In other words, the greater one’s PSMU, the greater one’s intentions to click, like, comment, and share news posts, agnostic to the type of content. When examining these relationships with respect to type of content, we found a significant relationship between PSMU and intent to click on false news, and a near-significant relationship between PSMU and intent to share false news. To the best of our knowledge, the current study is the first to use a behavioral task to assess susceptibility to false news and relate it to a substance use or behavioral addictive disorder. That said, Guelmami and colleagues developed a novel scale to assess susceptibility to COVID-19 disinformation, finding that one’s score on this scale was positively related to participants’ degree of general problematic internet use [41]. Our current research aligns with this previous research and extends it by specifically investigating PSMU and susceptibility to false news in general through the use of widely-used, previously-established measures.

Our results are important for several reasons. To begin with, it appears that the individuals who experience the most distress and impairment in daily functioning from social media use are also the most susceptible to the false information posted on social media. Clinicians and therapists will benefit from knowing that individuals displaying PSMU are more likely to be susceptible to online misinformation. This is relevant to treatment, for example, if an individual displaying PSMU goes online to obtain information about PSMU and finds misinformation. In support of this, a recent study demonstrated that the veracity of online information about “social media addiction” varied greatly when searching online [42]. Importantly, individuals who display PSMU may also be more susceptible to other types of health-related misinformation (e.g., COVID-19, vaccines, etc.). So, clinicians specializing in these different fields may also benefit from awareness of their patients’ degree of PSMU. Finally, there have been calls in academia [21], along with efforts by governments around the world [43], to stop the flow of false information online. Social media platforms like X (formerly Twitter) have also taken steps to combat misinformation, such as through their community notes program [44]. Therefore, our results are important for these endeavors, as knowing which individuals are more susceptible to fake news, can aid in stopping the spread and impact of misinformation. For example, researchers could potentially collaborate with social media platforms to help identify individuals displaying PSMU and augment their experience on these platforms to reduce the dissemination and effects of fake news.

Despite the insights provided by the above-described research, our study has limitations that we would like to mention. Primarily, we did not pretest our stimuli to control for aspects that could differ between the fake news stimuli and the real news stimuli. For example, previous research has revealed that belief in fake news can be induced by eliciting emotions [45], yet we did not pretest our stimuli for potential emotions elicited [46] or collect data in regard to this with our sample. Therefore, we allowed emotional responses to posts in our task to potentially vary between participant and condition. In addition, our real and fake news stories were taken from the internet, and it could be that the fake news stories circulated longer than the real news stories, and as a result, study participants could have been more familiar with the fake news stories. Indeed, previous research has demonstrated that familiarity is correlated with accuracy in identifying fake news [28]. Furthermore, as mentioned in the Methods section, we purposely did not balance news article topics or sources (web addresses) between conditions. Overall, our lack of pretesting and subsequent controlling for these factors could impact the interpretation of our findings. To explain, the observed significant differences between the real news and fake news conditions could be the result of our intended news-type manipulation, alternately however, our significant effects could also be due to stimuli differences in the above-mentioned aspects. We would like to note, however, that our research design was purposeful. We conducted a within-subject experiment to control for individual differences, and we specifically did not balance stimuli topics or sources to avoid disrupting the face validity of our manipulation – we wanted participants to be able to use the source of the news story in their credibility judgments. Nevertheless, we did not control for potential stimulus level differences. Therefore, although our results may be provocative, future research should be conducted to determine the exact mechanisms driving the presented findings.

Our study has other limitations that also deserve mention. First, we conducted our experiment with a convenience sample of undergraduate college students, so our study’s generalizability to other populations is limited. However, given the high prevalence of both general social media use [47] and PSMU [15] in this demographic, understanding the reported phenomena in this particular group is important. Next, we employed attention checks and a survey-seriousness assessment, adhering to published recommendations for employing attention checks in research [48], however, we did not validate our measures. As a result, we may have removed participants who were indeed paying attention to the experiment and taking it seriously. Finally, our study did not address causality. It could be that repeated exposure to sensational, false news may contribute to PSMU in certain individuals. Conversely, it could be that individuals are not predisposed to be susceptible to misinformation and developing PSMU leads to a greater susceptibility to misinformation. Future research will likely be able to investigate this potential causality, providing a better understanding of PSMU and potential targets for therapeutic interventions.

In sum, we found that the greater one’s PSMU, the more one finds false news credible. We also found that the greater one’s PSMU, the more one engages with posts of news articles on social media, as measured by participants’ intention to click, like, comment, and share news posts, regardless if the post consisted of real or false news. Importantly, our study also revealed that the greater one’s PSMU, the more one intends to click on posts specifically containing fake news. We also observed a strong trend between PSMU and one’s intention to share fake news posts. Future research will likely build upon these findings, such as examining different types of misinformation (e.g., political news), and potential interventions to stop the influence of misinformation on individuals displaying PSMU.
